# Factors Affecting the Accumulation of Curcumin in Microrhizomes of *Curcuma aromatica* Salisb

**DOI:** 10.1155/2015/740794

**Published:** 2015-02-02

**Authors:** Ke Wu, Xiaoxia Zhang, Shulan Sun, Xiaojing Wang

**Affiliations:** Guangdong Key Lab of Biotechnology for Plant Development, College of Life Sciences, South China Normal University, Guangzhou, Guangdong 510631, China

## Abstract

Curcuminoids, and mainly curcumin, are potential therapeutic agents for the prevention of various diseases; however, little is known about the factors that influence their accumulation in *Curcuma* species. In this study, the effects of factors such as sucrose concentration, different ratios of 6-benzylaminopurine (6-BA) and *α*-naphthalene acetic acid (NAA), and light quality on the accumulation of curcumin and other curcuminoids in *Curcuma aromatica* were investigated. Microrhizomes grown on media containing 3% sucrose produced more curcumin and other curcuminoids than those grown on higher concentrations. Moreover, when compared to other ratios of 6-BA and NAA, microrhizomes induced on 3% sucrose media supplemented with 3.0 mg/L 6-BA and 0.5 mg/L NAA produced more curcumin and other curcuminoids; however, the amount was less than in microrhizomes grown on 3% sucrose alone. We determined that a 5% sucrose medium supplemented with 3.0 mg/L of 6-BA and 0.5 mg/L of NAA enhanced the levels of curcumin and curcuminoids and that exposure to red light further increased production.

## 1. Introduction

Rhizomes of* Curcuma* species are used as spices, flavoring and coloring agents, and traditional medicines in Asian countries. Recent studies have shown that* Curcuma aromatica* exhibits various pharmacological properties, including anti-inflammatory [1], antioxidant [2], antiangiogenic [3], and anticarcinogenic [4] effects that have been attributed to the presence of secondary metabolites. Polyphenolic curcuminoid compounds including curcumin, bisdemethoxycurcumin, and demethoxycurcumin are the main secondary metabolites in* Curcuma longa* and other* Curcuma* species and, of these, curcumin is the best studied and has been shown to exhibit anti-inflammatory, anticarcinogenic, and antioxidant activities [5–7]. Consequently, curcumin has received considerable interest as a potential therapeutic agent for the prevention of various diseases [8]; however, little is known about the factors that influence its accumulation in* Curcuma* species.

Recently, it has been discovered that microrhizomes can develop* in vitro* in a growth medium with increased sucrose levels and containing plant growth regulators (PGRs) such as 6-benzylaminopurine (6-BA) and *α*-naphthalene acetic acid (NAA). Furthermore, larger microrhizomes are capable of survival in the field without any specific acclimatization procedure after this induction [9–11]. Microrhizomes were also found to produce secondary metabolites with antioxidant activity equaling or surpassing commercial dried powdered rhizome preparations of field-grown plants [12].* In vitro* regeneration systems are often useful for the production of phytomedicine since conventionally cultivated plants can suffer from exposure to a variety of pests, weather conditions, and land availability, which can adversely affect the medicinal qualities of the harvested plants [13]. It is, therefore, of great interest to identify factors that affect the accumulation of curcumin in induced microrhizomes grown under controlled conditions* in vitro*.

The biosynthetic pathway of curcumin begins with phenylalanine, which is a common precursor in the biosynthesis of flavonoids [14, 15]. Phenylalanine ammonia lyase (PAL) catalyses the first committed step in the biosynthesis of phenolic compounds and is involved in responses to a variety of biotic and abiotic stresses [16]. Increased PAL activity has been reported to promote the subsequent reactions in the phenylpropanoid pathway, thereby leading to the increased production of phenylpropanoid derivatives, including phenols and flavonoids [17]. In this context, a variety of different elicitors can regulate the production of secondary metabolites, which are often used in plant defense and stress tolerance [18]. For example, studies have revealed that PGRs such as 2,4-dichlorophenoxy acetic acid (2,4-D) or NAA affect the expression of PAL and the accumulation of phenolic compounds in some plant species. For example, application of gibberellin (GA_3_), NAA, and 2,4-D to wax apple (*Syzygium samarangense*) enhanced PAL and antioxidant activities, which were positively correlated with increases in phenolic and flavonoid levels [19]. It has also been reported that the PGRs NAA and thidiazuron (TDZ) affect the content of phenolic compounds, flavonoids, and their antioxidant activity in callus cultures of* Artemisia absinthium* L. [20]. Additionally, light is known to regulate not only plant growth and development, but also the biosynthesis of primary and secondary metabolites [21, 22]. As an example, different light intensities can affect total phenolic levels, flavonoid synthesis, and antioxidant activities in young ginger (*Zingiber officinale*) varieties [23].

To our knowledge, the effect of light quality on the biosynthesis of curcumin or other phenolic compounds by members of the Zingiberaceae family has not been reported, although it is known from studies of other plant taxa that light quality can affect the synthesis of such compounds. For example, exposure to red light can result in an increased accumulation of total phenolic compounds, flavonols, and flavonoids, as well as greater PAL enzymatic activity and transcript levels [24]. While red light has also been found to have a stimulatory effect on the phenylpropanoid pathway in* Ocimum basilicum* [25], it has been reported that light in the green spectrum enhances the production of phenolic compounds, flavonoids, and antioxidant activity in callus cultures of* A. absinthium* [20]. In addition, in the context of microrhizome formation, sucrose concentration has been reported to be a major factor in several* Curcuma *species [9–11].

Taken together, the results of these studies led us to speculate that sucrose concentration, PGRs, and light quality may influence the production of curcumin and other curcuminoids, a hypothesis that we address here experimentally. The present study was undertaken in an effort to establish an efficient system for microrhizome induction, growth of microrhizomes, and the production of curcumin under controlled conditions, thereby maximizing the total yield of curcumin.

## 2. Material and Methods

### 2.1. Plant Material

*Curcuma aromatica* rhizomes were collected from wild plant populations and planted in a plantation center at the South China Normal University. Mature rhizomes collected from November to December 2011, a gift from Professor Yingqiang Wang from the South China Normal University, were cleaned thoroughly under running tap water and dormant buds measuring 1-2 cm with small portions of attached rhizome were then excised.

### 2.2. Culture Conditions

The growth medium was solidified with 0.75% agar and autoclaved before the pH was adjusted to 5.8. Except for treatments involving different light quality, cultures were maintained in a growth chamber at 25 ± 2°C under a 16/8-h (light/dark) white fluorescent light photoperiod with a photosynthetic photon flux of 60 *μ*mol/m^2^/s and relative humidity of 40 ± 5%.

### 2.3. Shoot Induction and Multiplication

Dormant buds were treated with 75% ethanol for 1 min under aseptic conditions, before being sterilized with a 0.1% HgCl_2_ solution for 15 min and subsequently rinsed 7 times with sterile distilled water. Surface sterilized buds were placed on bud initiation medium (MS supplemented with 2.0 mg/L 6-BA and 0.5 mg/L of NAA) for approximately 30 d. Emerged young buds were dissected into single buds for subculturing onto fresh bud initiation medium after 30 d. The clustered buds were cut into single buds, the leaves and roots were excised, and the shoot base was cut into 4 parts, which were used as explants for subsequent analysis. The explants were inoculated onto Murashige and Skoog (MS) medium containing 3.0 mg/L of 6-BA and 0.5 mg/L of NAA. Plantlets formed from these explants were subcultured onto fresh medium for subsequent multiplication.

### 2.4. Induction of Microrhizomes

To study the effects of plant growth factors and sucrose in different combinations and concentrations on the induction of microrhizomes, clustered buds derived from aseptic cultures were cut into single buds and the leaves and roots were excised, leaving approximately 0.5 cm of shoot base, which was placed on one of the following 14 combinations of media for microrhizome induction:

 CM1: MS + sucrose 30 g/L,
 CM2: MS + sucrose 50 g/L,
 CM3: MS + sucrose 70 g/L,
 CM4: MS + sucrose 90 g/L,
 CM5: MS + sucrose 30 g/L + 6-BA 1.0 mg/L + NAA 0.1 mg/L,
 CM6: MS + sucrose 30 g/L + 6-BA 3.0 mg/L + NAA 0.1 mg/L,
 CM7: MS + sucrose 30 g/L + 6-BA 5.0 mg/L + NAA 0.1 mg/L,
 CM8: MS + sucrose 30 g/L + 6-BA 1.0 mg/L + NAA 0.5 mg/L,
 CM9: MS + sucrose 30 g/L + 6-BA 3.0 mg/L + NAA 0.5 mg/L,
 CM10: MS + sucrose 30 g/L + 6-BA 5.0 mg/L + NAA 0.5 mg/L,
 CM11: MS + sucrose 30 g/L + 6-BA 3.0 mg/L + NAA 0.5 mg/L,
 CM12: MS + sucrose 50 g/L + 6-BA 3.0 mg/L + NAA 0.5 mg/L,
 CM13: MS + sucrose 70 g/L + 6-BA 3.0 mg/L + NAA 0.5 mg/L,
 CM14: MS + sucrose 90 g/L + 6-BA 3.0 mg/L + NAA 0.5 mg/L.

After 30 days, 60 plantlets were individually subcultured onto fresh medium divided by 10 sterile growth containers for every treatment. After 90 days, the weight and number of microrhizomes formed per explant were noted and the induction rate was calculated using the following formulas: induction rate of microrhizomes = number of microrhizomes/explant × 100(%).

### 2.5. Light Treatments

Clustered buds were cut into single buds, the leaves and roots were excised, and approximately 0.5 cm of shoot base was placed onto MS + 5% Suc + 3.0 mg/L 6-BA + 0.5 mg/L NAA. For each treatment, there were 10 containers with 6 explants and the culture conditions were as described above. Five light quality treatments were investigated: (1) fluorescent light (white fluorescent lamps), (2) red LED (wavelength: 625 ± 5 nm), (3) blue LED (wavelength: 425 ± 5 nm), (4) red plus blue (7 : 3 photon flux density) LED, and (5) red plus blue (3 : 7 photon flux density) LED. The LED system was obtained from Royal Philips (Netherlands) with a 60 *μ*mol/m^2^/s light intensity.

### 2.6. Extraction of Curcumin

Microrhizomes were sliced and dried at 60°C for 48 h and then powdered. 1.5 mL of 80% ethanol was added to 50 mg of dried rhizomes and refluxed for 2 h at 30°C in the dark. This mixture was then centrifuged at 5000 g for 15 min and filtered through a 0.25 *μ*m membrane filter and the filtrate was analyzed by HPLC.

### 2.7. Determination of Curcumin Levels

HPLC analysis of curcumin and demethoxycurcumin content was performed as described by Zhan et al. [26], using a Shimadzu SPD-20A HPLC system (Shimadzu, Japan) with a LC-20AT UV detector, a YMC-packed ODS column (250 mm × 4.6 mm, 5 *μ*m), and associated analytical software. The mobile phase was acetonitrile: 5% acetic acid aqueous solution (50 : 50, v/v); the temperature of column was 30°C and the UV detection wavelength was 425 nm. All samples were filtered through 0.25 *μ*m membrane filters before injection into the HPLC. The curcumin peak in the samples was identified by its retention time and a coinjection test with standard curcumin. Quantitative analysis was performed using the peak area based on a standard curve.

### 2.8. Statistical Analyses

Statistical analyses were conducted with the Statistical Products and Service Solutions for Windows, version 19.0 (SPSS, Japan). The difference between means was tested using Duncan's Multiple Range Test at *P* < 0.05. All experiments were repeated at least three times and the results are given as the mean of three independent experiments ± standard error.

## 3. Results

### 3.1. Influence of Sucrose on Production of Curcuminoids

**Figure 1:**
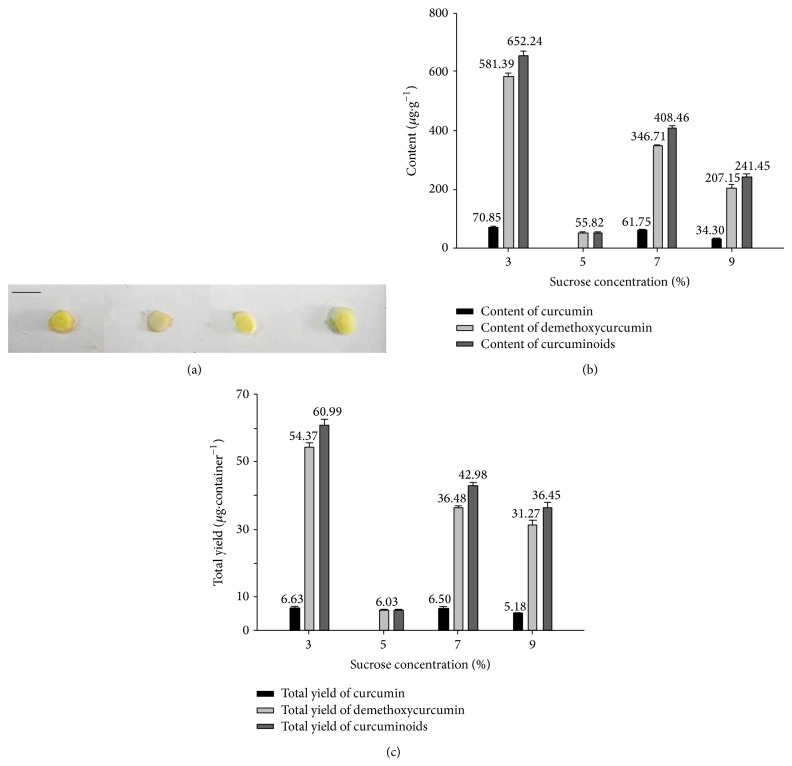
Effect of sucrose concentration on accumulation of curcumin and curcuminoids in induced microrhizomes. (a) Transection of induced microrhizomes on MS containing different concentrations of sucrose from 3% to 9% (left to right). Scale bar = 1 cm. (b) Content of curcumin and curcuminoids in dry microrhizomes. (c) Total yield of curcumin and curcuminoids in individual containers of microrhizomes.

Four different concentrations of sucrose were included in the MS media to examine the effect on the production of curcumin. The color of the microrhizomes grown on MS varied with sucrose concentration (Figure 1(a)) and trichomes grown on 5% sucrose were almost white, indicating the absence or low levels of curcumin, which is yellow. HPLC analysis supported this observation and revealed that curcumin and other curcuminoids were barely detectable in rhizomes grown on 5% sucrose, while an increase in sucrose from 3% to 7% or 9% resulted in reduced production of these compounds (Figure 1(b)). Interestingly, 5% sucrose concentration was most effective for microrhizome induction with a 79% induction rate compared to the lowest rate for MS containing 3% sucrose, which was 36% (Table 1). The weight of microrhizomes grown on MS containing 5% and 3% sucrose was 0.0233 g and 0.0423 g (Table 1), respectively; however, when the number and weight of microrhizomes were taken into account, MS containing 3% sucrose resulted in the highest total yield of curcumin, demethoxycurcumin, and curcuminoids (Figure 1(c)).

### 3.2. Influence of Plant Growth Factors on the Production of Curcuminoids

Based on the above data, it was established that an MS medium with 3% sucrose was the most effective for the production of curcumin. We then assessed the effects of including different combinations of plant growth factors on the number and weight of microrhizomes. Generally, plant growth factors were found to have substantial effect on the number of microrhizomes produced (Table 2). When the concentration of 6-BA was increased in MS containing 3% sucrose, the number of produced microrhizomes decreased, except for the combination of 3.0 mg/L of 6-BA with 0.5 mg/L of NAA. In contrast to 6-BA, NAA had little effect on the production of microrhizomes other than the combination of 3.0 mg/L of 6-BA with 0.5 mg/L of NAA. Interestingly, although supplementing the MS medium with either 6-BA or NAA alone did not have any effect, a combination of 3.0 mg/L of 6-BA with 0.5 mg/L of NAA increased the production of microrhizomes significantly. Regarding the weight of the produced microrhizomes, the different concentrations of 6-BA or NAA alone or in combination did not have any significant effect. Taken together, MS medium containing a combination of 3.0 mg/L of 6-BA and 0.5 mg/L of NAA was found to be optimal for the production of* C*.* aromatica*. microrhizomes.

However, the levels of curcumin, demethoxycurcumin and curcuminoids were greatest in the microrhizomes grown on MS medium containing 3.0 mg/L of 6-BA with 0.1 mg/L NAA, and indeed were more than twice those in microrhizomes grown in MS mediumcontaining 3.0 mg/L of 6-BAand 0.5 mg/L NAA. It should be noted that if the number and weight of microrhizomes are taken into account, the yield of curcumin, demethoxycurcumin, and curcuminoids was maximal in microrhizomes grown in MS medium containing 3.0 mg/L of 6-BA with 0.5 mg/L NAA (Table 2).

### 3.3. Effect of the Combination of Plant Growth Regulators and Concentration of Sucrose on Production of Curcuminoids

Although microrhizomes grown on MS containing 3% sucrose supplemented with the optimal combination of plant growth regulators produce the most curcumin, the levels were still lower than those of microrhizomes grown on MS containing 3% sucrose alone. Therefore, the optimal combination of PGRs (3.0 mg/L of 6-BA with 0.5 mg/L NAA) was combined with different concentrations of sucrose to evaluate their overall influence on the yield of curcuminoids. As shown in Table 3, the number of induced microrhizomes and their weight were highest with 5% sucrose and the optimal combination of PGRs. However, the accumulation of curcumin did not follow the same trend since the content gradually increased from 8.03 *μ*g/g to 89.66 *μ*g/g with an increase in sucrose concentration from 3% to 7% and then decreased to 32.49 *μ*g/g with 9% sucrose in the medium. The content of demethoxycurcumin increased substantially from 198.13 *μ*g/g to 558.89 *μ*g/g between sucrose concentrations of 3% and 5% and then showed a slight decrease to 517.03 *μ*g/g with 7% sucrose and major reduction to 289.08 *μ*g/g with 9% sucrose. Taken together, the content of curcuminoids was highest in 5% sucrose containing MS supplemented with 3.0 mg/L of 6-BA and 0.5 mg/L NAA, but the levels were not significantly different from those of the 7% sucrose grown microrhizomes. When factors such as the frequency of rhizome formation and their weight were also considered, the production of curcuminoids was far higher with the 5% sucrose media and particularly when compared to the media with 7% sucrose.

### 3.4. Influence of Light Quality on Production of Curcuminoids

We observed that light quality plays a significant role in the accumulation of curcuminoids in microrhizomes grown on 5% sucrose supplemented media with 3.0 mg/L of 6-BA and 0.5 mg/L NAA. Compared with the white light treatment, the number of induced microrhizomes was lower under all other light types. The number of induced microrhizomes was highest under red light (60%) and lowest under blue light (38%). Similarly, the weight of the produced microrhizomes was highest and lowest when grown under red or blue light, respectively. However, overall the light quality only slightly affected the weight of the microrhizomes, which decreased under all lights treatments compared to under white light. The content of curcumin and demethoxycurcumin in microrhizomes grown under red light was the highest among the different tested light conditions. Levels decreased when blue light was added and the lowest levels of curcumin and demethoxycurcumin were detected in microrhizomes grown under blue light alone. In contrast, the content of curcumin and demethoxycurcumin in microrhizomes grown under 7/10 red light mixed with 3/10 blue light was similar to that in microrhizomes grown under white light. We, therefore, concluded that the optimal light for production of curcuminoids is red light.

## 4. Discussion

The induction of microrhizomes has been widely studied in* Curcuma* species, but the factors that affect the accumulation of the main secondary metabolite, curcumin, have not been identified. In the present study, three factors thought to affect the production of curcumin were investigated in* C*.* aromatica*.

PGRs have long been used to induce callus or shoots from explants and the effects of phytohormones on plant growth and development have been extensively studied. Importantly, IAA and 6-BA have been reported to promote the induction of microrhizomes of several* Curcuma* species [9, 27]. In our study, we found that an appropriate combination of sucrose and PGRs resulted in an increase in the accumulation of curcumin. Specifically, the amount of curcumin in microrhizomes grown on MS containing 3% sucrose was 70.85 *μ*g/g (Figure 1(b)) but decreased to 55.82 *μ*g/g in microrhizomes grown on MS containing 3% sucrose with 3.0 mg/L of 6-BA and 0.1 mg/L of NAA (Table 2). However, if grown on MS containing 7% sucrose supplemented with 3.0 mg/L of 6-BA and 0.5 mg/L of NAA the curcumin content was 89.66 *μ*g/g (Table 3). This indicates that different combinations of sucrose and PGRs substantially affect the curcumin content.

Plant secondary metabolites have a range of important biological and pharmacological activities, such as antioxidative and anticarcinogenic [28–30], and the biological activities of phenolic compounds and flavonoids are associated with their antioxidant potential [31]. Some stresses, such as ultraviolet B (UV-B) light, can cause an increase in the levels of phenolic compounds, which protect plants against injury and function as an antioxidant system [32]. This is congruent with the observation that treatment of wax apple with PGRs resulted in higher PAL and antioxidant activity levels and that specific combinations of GA3, NAA, and 2, 4-D are positively correlated with increases in the levels of phenolic compounds and flavonoids [19]. PGRs, such as NAA and TDZ, have been reported to affect the content of phenolic compounds, flavonoids, and antioxidant activity in callus cultures of* A. absinthium* L. [20] and auxin application enhances the total polyphenolic content and nutritional value of grape [33]. Moreover, the application of exogenous 2,4-epibrassinolide to grape berries has been shown to enhance the content of phenolic compounds, as well as the antioxidant potential and PAL activity [34]. Our results are consistent with the idea that PGRs play an important role in the accumulation of phenolic compounds, which may be due to the fact that PGRs can promote the production of reactive oxygen species (ROS), which in turn induces the antioxidant system, including the accumulation of phenolic compounds and flavonoids. This might also suggest that an increase in curcumin levels may coincide with higher PAL activity and antioxidant activity levels, as a consequence of PGR action, although this has yet to be tested experimentally.

It has been proposed that members of Zingiberaceae family transport antioxidants to their rhizomes, where they accumulate [35]. Additionally, rhizome dry matter accumulation increases at the expense of leaf and root growth as rhizomes become competitive sinks. Cousins et al. [12] found that the rhizomes of turmeric (*C. longa*) have higher relative dry weight (dry weight/fresh weight) values than other organs, indicating an active accumulation of carbohydrates in these storage organs. The authors also found a linear relationship between dry weight and sugar use and suggested that this information will be useful in the development of a model for the conversion of sugars into biomass and subsequently into secondary metabolites [36]. Appropriate PGR treatments can increase the total sugar content of some fruits [37] and may influence the source/sink balance in the plant, resulting in an increase in the rate of carbohydrate accumulation [19]. Our results demonstrated that PGR treatments can increase curcumin levels in* C. aromatica* and suggest that the appropriate sucrose concentration in the medium may supply sugar for secondary metabolite production. A critical factor in the accumulation of curcumin is the balance of factors such as a high sucrose concentration and addition of PGRs, which may affect source sink-relationships and promote effective partitioning of carbon to curcuminoids.

Light quality was also found to greatly affect the accumulation of curcumin. When microrhizomes were grown under red light in MS containing 5% sucrose, 3.0 mg/L 6-BA, and 0.5 mg/L NAA, the curcumin content increased to 107.34 *μ*g/g compared to 74.36 *μ*g/g in microrhizomes grown under white light (Table 4). Although the effect of light quality on curcumin production has not previously been described, it has been reported that light quality affects the formation of phenolic compounds. Shiga et al. [25] reported that red light has a stimulatory effect on the phenolic pathway in* Ocimum basilicum* [25], and this conclusion was supported by the discovery that the accumulation of phenolic compounds, flavonols, and flavonoids, as well as PAL activity and transcript levels, increased when* Linum album* cell cultures were exposed to red light [24]. However, callus cultures of* A. absinthium* were found to have higher levels of phenolic compounds, flavonoids, and antioxidant activity when grown under a green light [20]. In this current study, red light effectively increased the production of curcumin; however, the underlying mechanism remains unknown. One hypothesis is that red light triggers the phytochrome mediated signaling pathway. Light quality has been shown to affect not only the accumulation of polyphenols but also their subsequent antioxidant activity, as in the case of* Inonotus obliquus* grown in submerged cultures, which were exposed to a range of different light conditions [38]. The authors revealed that continuous darkness enhanced the formation of polyphenols with lower levels in mycelia grown under blue and red light and that polyphenols synthesized during daylight showed less potential antioxidant activity than those synthesized under other light regimes [38]. Therefore, it will be of great interest to further investigate the expression of components of the light signaling pathway and enzymes responsible for biosynthesis of curcumin, such as PAL and curcumin synthase (CURS) [39], and the subsequent antioxidant activity of curcumin.

In addition to the yield of curcumin, curcumin dry weight amounts were assessed by taking into account the number and weight of the induced microrhizomes and the content of demethoxycurcumin. The total yield of curcuminoids of microrhizomes grown in MS containing 3% sucrose, 3.0 mg/L 6-BA, and 0.5 mg/L NAA was 24.73 *μ*g in each container (Table 2). However, the total yield of curcuminoids was substantially increased (80.22 *μ*g per container) when microrhizomes were grown in MS containing 5% sucrose, 3.0 mg/L 6-BA, and 0.5 mg/L NAA (Table 3). Moreover, the total yield of curcuminoids increased slightly (83.93 *μ*g per container) when grown under red light (Table 4). We observed that the number and weight of the induced microrhizomes were much higher when grown under white light than under red light (Table 4).

The study described here provides new insights into the manipulation of curcuminoid levels in* C*.* aromatica* microrhizomes using specific light regimes and combinations of sucrose and PRGs. That is, 5% sucrose medium supplemented with 3.0 mg/L of 6-BA and 0.5 mg/L of NAA and exposure to red light enhanced greatly the levels of curcumin in microrhizomes of* Curcuma aromatica*. We conclude that different combinations of sucrose and PRGs and light exposure may help optimize growth and developmental changes of microrhizomes and that this may help in the practical application of phototechnology with respect to design of a controlled environment for* in vitro* curcuminoid production.

## Figures and Tables

**Table 1:** Effect of sucrose concentration on *Curcuma aromatica* Salisb. microrhizome production.

Sucrose concentration (%)	Induction rate of microrhizomes (%)	Average weight of microrhizomes (g)
3	35.73 ± 0.95	0.0423 ± 0.0005
5	78.75 ± 0.42	0.0233 ± 0.0003
7	52.84 ± 2.84	0.0332 ± 0.0017
9	64.17 ± 2.50	0.0418 ± 0.0010

**Table 2:** Effect of 6-BA and NAA in MS containing 3% sucrose on production of microrhizomes and curcuminoids.

MS + 3% (w/v) sucrose + plant growth regulator (mg/L)		Induction rate of microrhizomes (%)	Average weight of microrhizomes (g)	Content of curcumin (*μ*g/g)	Content of demethoxycurcumin (*μ*g/g)	Content of curcuminoids (*μ*g/g)	Total yield of curcumin (*μ*g/bottle)	Total yield of demethoxycurcumin (*μ*g/bottle)	Total yield of curcuminoids (*μ*g/container)
NAA	6-BA								
0.1	1.0	55.67 ± 1.00^b^	0.02342 ± 0.0035^a^	ND	ND	ND	ND	ND	ND
0.1	3.0	25.00 ± 1.67^d^	0.02341 ± 0.0118^a^	534.98 ± 0.29^a^	397.69 ± 1.23^a^	3452.67 ± 1.52^a^	1.99 ± 0.01^b^	134.38 ± 0.034^c^	16.36 ± 0.05^c^
0.1	5.0	31.67 ± 1.67^c^	0.0190 ± 0.0053^a^	15.340 ± 1.33^c^	165.28 ± 18.13^c^	180.69 ± 19.346^c^	0.55 ± 0.05^d^	5.95 ± 0.65^d^	6.51 ± 0.70^d^
0.5	1.0	61.67 ± 5.00^b^	0.0228 ± 0.00348^a^	18.28 ± 1.91^bc^	183.13 ± 34.834^c^	201.341 ± 6.76^c^	1.534 ± 0.16^c^	15.348 ± 0.341^b^	17.02 ± 0.57^b^
0.5	3.0	76.67 ± 6.67^a^	0.02346 ± 0.0080^a^	20.27 ± 0.349^b^	198.13 ± 34.65^b^	218.341 ± 5.134^b^	2.30 ± 0.06^a^	22.3434 ± 0.53^a^	234.73 ± 0.58^a^
0.5	5.0	36.67 ± 3.334^c^	0.01734 ± 0.0033^a^	ND	ND	ND	ND	ND	ND

Values followed by different letters differ significantly. *P* < 0.05. ND: not detected.

**Table 3:** Effect of sucrose concentration in MS supplemented with 3.0 mg/L 6-BA and 0.5 mg/L NAA on production of microrhizomes and curcuminoids.

Sucrose concentration (%)	Induction rate of microrhizomes (%)	Average weight of microrhizomes (g)	Content of curcumin (*μ*g/g)	Content of demethoxycurcumin (*μ*g/g)	Content of curcuminoids (*μ*g/g)	Total yield of curcumin (*μ*g/container)	Total yield of demethoxycurcumin (*μ*g/container)	Total yield of curcuminoids (*μ*g/container)
3	78.33 ± 5.00^b^	0.0226 ± 0.0014^a^	8.03 ± 2.05^d^	198.13 ± 4.65^c^	206.16 ± 6.70^c^	0.85 ± 0.22^c^	21.06 ± 0.49^d^	21.92 ± 0.71^d^
5	90.00 ± 2.00^a^	0.0235 ± 0.0019^a^	74.36 ± 4.32^b^	558.89 ± 24.97^a^	633.25 ± 29.29^a^	9.42 ± 0.55^a^	70.80 ± 3.16^a^	80.22 ± 3.71^a^
7	80.33 ± 1.67^b^	0.0211 ± 0.0009^a^	89.66 ± 6.25^a^	517.03 ± 36.08^a^	606.69 ± 42.32^a^	9.12 ± 0.64^a^	52.58 ± 3.67^b^	61.70 ± 4.30^b^
9	81.00 ± 0.33^b^	0.0228 ± 0.0027^a^	32.49 ± 0.24^c^	289.08 ± 38.45^b^	321.57 ± 38.68^b^	3.60 ± 0.03^b^	32.06 ± 4.26^c^	35.66 ± 4.29^c^

Values followed by different letters differ significantly. *P* < 0.05.

**Table 4:** Effect of light quality on accumulation of curcumin and curcuminoids in induced microrhizomes.

Light quality	Induction rate of microrhizomes (%)	Average weight of microrhizomes (g)	Content of curcumin (*μ*g/g)	Content of demethoxycurcumin (*μ*g/g)	Content of curcuminoids (*μ*g/g)	Total yield of curcumin (*μ*g/container)	Total yield of demethoxycurcumin (*μ*g/container)	Total yield of curcuminoids (*μ*g/container)
White light	90.00 ± 2.00^a^	0.0235 ± 0.0019^a^	74.36 ± 4.32^bc^	558.89 ± 24.97^b^	633.25 ± 58.57^b^	9.42 ± 0.55^a^	70.80 ± 3.16^a^	80.22 ± 3.71^a^
Red light	60.00 ± 5.45^b^	0.0241 ± 0.0003^a^	107.34 ± 4.29^a^	854.60 ± 7.20^a^	961.94 ± 10.13^a^	9.56 ± 0.09^a^	74.37 ± 0.55^a^	83.93 ± 0.65^a^
Red light : blue light = 7 : 3	50.00 ± 2.72^c^	0.0228 ± 0.0002^b^	83.57 ± 0.82^b^	616.73 ± 2.78^b^	700.31 ± 2.19^b^	5.71 ± 0.07^c^	42.20 ± 0.18^b^	47.90 ± 0.25^b^
Red light : blue light = 3 : 7	57.78 ± 4.16^b^	0.0223 ± 0.0002^c^	87.22 ± 1.19^b^	446.47 ± 8.52^c^	533.69 ± 8.17^c^	6.78 ± 0.10^b^	34.09 ± 0.15^c^	40.87 ± 0.25^c^
Blue light	37.78 ± 4.16^c^	0.0218 ± 0.0004^bc^	70.25 ± 1.23^c^	362.83 ± 4.50^d^	433.08 ± 5.68^d^	3.45 ± 0.05^d^	17.83 ± 0.12^d^	21.27 ± 0.18^d^

Values followed by different letters differ significantly. *P* < 0.05.
